# A Case of Horseshoe Kidney and Autosomal Dominant Polycystic Kidney Disease with *PKD1* Gene Mutation

**DOI:** 10.3390/jcm14114008

**Published:** 2025-06-05

**Authors:** Hyeongwan Kim, Soo Jin Lee, Won Kim

**Affiliations:** 1 Department of Internal Medicine, Jeonbuk National University Medical School, Jeonju 54907, Republic of Korea; ocaju78@gmail.com (H.K.); 423-soojin@hanmail.net (S.J.L.); 2 Research Institute of Clinical Medicine of Jeonbuk National University-Biomedical Research Institute of Jeonbuk National University Hospital, Jeonju 54907, Republic of Korea

**Keywords:** polycystic kidney disease, horseshoe kidney, *PKD1* gene

## Abstract

**Background/Objectives**: Horseshoe kidney is a congenital anomaly characterized by the fusion of the kidneys at the lower pole. Polycystic kidney disease with horseshoe kidney is called polycystic horseshoe kidney. Genetic testing is essential for the diagnosis of polycystic horseshoe kidney disease because it can result from a number of genetic disorders. Fewer than 20 cases of polycystic horseshoe kidney have been reported to date. However, polycystic horseshoe kidney disease was mostly diagnosed via autopsy or radiologic imaging techniques including computed tomography, magnetic resonance imaging, and angiography. Because polycystic kidney disease has various causes, genetic testing is essential for the diagnosis of autosomal dominant polycystic kidney disease (ADPKD) in patients with polycystic horseshoe kidney disease. At present, the diagnosis of ADPKD is made using genetic approaches, including next-generation sequencing. We reported a potentially pathogenic polycystin 1 (*PKD1*) gene in a patient with ADPKD and horseshoe kidney. **Methods**: We performed the sequencing of the *PKD1* gene and radiological examinations (computed abdominal tomography). **Results**: Computed abdominal tomography revealed enlarged kidneys with multiple cysts fused at the lower poles, indicating polycystic HSK. The sequencing of the *PKD1* gene revealed a heterozygous pathogenic variant c.165_171del (p.Leu56ArgfsTer15), which genetically confirmed the diagnosis of ADPKD. The patient was treated with an angiotensin II receptor blocker. **Conclusions**: In this case report, we suggest that genetic testing becomes the key approach to the diagnosis of ADPKD with horseshoe kidney. Additionally, this approach offers the benefit of avoiding the possibility of the condition being mistakenly diagnosed or diagnosed late due to its uncommon occurrence and nonspecific symptoms.

## 1. Introduction

Polycystic kidney disease can arise from various genetic diseases, including autosomal dominant polycystic kidney disease (ADPKD) [1], autosomal recessive polycystic kidney disease [2], autosomal dominant tubulointerstitial kidney disease [3], and nephronophthisis [4]. ADPKD is a common hereditary disorder caused by mutations in the genes encoding polycystin 1 (*PKD1*) or polycystin 2 (*PKD2*). Autosomal recessive polycystic kidney disease is primarily caused by mutations in the ***PKHD1*** or *DZIP1L* gene [5]. Autosomal dominant tubulointerstitial kidney disease can be associated with defects in the *UMOD*, *MUC1*, *REN*, *HNF1B*, *SEC61A1*, and *DNAJB11* genes [3]. Nephronophthisis can be linked to abnormalities in the *NPHP* genes [6]. The reported prevalence of ADPKD ranges from 1 in 400 to 1 in 1000 [7,8,9,10].

Horseshoe kidney is a congenital anomaly characterized by the fusion of the kidneys at the lower pole, resulting in a U or horseshoe configuration [11]. This fusion transpires throughout fetal development, generally between the 7th and 9th weeks of gestation, when the kidneys are expected to migrate from the lower abdomen to their appropriate locations [12]. The prevalence of horseshoe kidney (HSK) ranges from 1 in 400 to 1 in 600 [13,14]. While several factors and associations have been identified, the exact genetic causes of horseshoe kidney remain unclear.

The coexistence of these distinct clinical conditions, referred to as polycystic HSK, is extremely rare [15]. Polycystic HSK is estimated to affect between 1 in 134,000 and 1 in 8,000,000 individuals, and fewer than 20 cases have been reported to date [15,16]. Because the causes of cystic kidney disease are diverse, genetic testing is essential for assessing the correlation between genetic abnormalities and polycystic HSK. However, most previous cases of PKD in polycystic HSK were identified through radiologic imaging techniques, including computed tomography (CT) and magnetic resonance imaging (MRI). The majority of patients do not receive a diagnosis through genetic testing. To the best of our knowledge, ADPKD with a mutation in the polycystin *1* or polycystin *2* gene in a patient with HSK has been reported only once [15].

In this study, the diagnosis of ADPKD is made using genetic approaches, including next-generation sequencing. We reported a potentially pathogenic polycystin 1 (*PKD1*) gene in a patient with ADPKD and horseshoe kidney.

## 2. Case Description

A 24-year-old male patient was referred to the nephrology outpatient clinic at Jeonbuk National University Hospital for a health checkup before joining the army. His father, aged 45 years, and his uncle, aged 46 years, were diagnosed with polycystic kidney disease and end-stage renal disease, requiring hemodialysis. The patient’s father and uncle did not undergo genetic testing and did not have horseshoe kidneys. The other five male siblings of the patient’s father did not have polycystic kidney disease, and the patient had a younger sister who refused to undergo testing. The patient had a four-year history of hypertension (150/90 mmHg). At that time, the patient was asymptomatic, and it was suggested that he had changed his lifestyle habits (diet and intense physical exercise).

**Figure 1:**
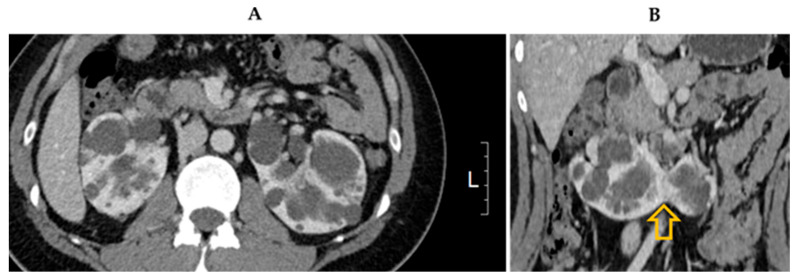
Computed tomography image showing (**A**) multiple bilateral cysts consistent with polycystic kidney disease and (**B**) kidneys fused at lower poles (arrow).

A physical examination of the abdomen revealed no palpable kidneys. The results of cardiovascular and respiratory system examinations were unremarkable. CT revealed enlarged kidneys with multiple cysts fused at the lower poles, indicating polycystic HSK (Figure 1). Additionally, multiple cysts were observed in the liver tissue. Brain magnetic resonance angiography revealed no evidence of an intracranial aneurysm or vascular malformation. Echocardiography revealed normal left ventricular systolic function (ejection fraction, 60%) with borderline left atrial enlargement.

The patient’s renal function was normal (0.85 mg/dL serum creatinine, 122 mL/min/1.73 m^2^ estimated glomerular filtration rate, 104 mL/min creatinine clearance, 16 mg/dL blood urea nitrogen, and 101.2 mL/min urea clearance), and there was no evidence of proteinuria or hematuria (urine examination pH 7.0, specific gravity 1.016, protein nil, sugar nil, ketones negative, red blood cells 0–2/HPF, and white blood cells 0–2/HPF). The urine protein-to-creatinine and albumin-to-creatinine ratios were 79 and 14 mg/g, respectively. Other investigations revealed fasting blood glucose levels of 76 mg/dL, serum sodium 138 mEq/L, serum potassium 4.6 mEq/L, alanine transaminase 38 U/L, aspartate transaminase 28 U/L, serum alkaline phosphatase 73 U/L, total bilirubin 0.74 mg/dL, serum proteins 7.2 g/dL, serum albumin 4.9 g/dL, hemoglobin 15.0 g/dL, total leukocyte count 7360/µL, differential cell count neutrophil 65%, lymphocyte 25%, monocyte 0.5%, eosinophil 0.1%, and platelet count 201,000/µL (Table 1).

Gene Panel Sequencing was performed. The genomic DNA extracted from the proband was analyzed using a customized hereditary polycystic kidney disease panel. This panel was designed to target the exons and adjacent regions of 34 genes associated with polycystic kidney disease, including ANKS6, CEP164, CEP83, COL4A1, DNAJB11, DZIP1L, GANAB, HNF1B, INVS, MAPKBP1, NPHP1, NPHP3, NPHP4, PKD1, PKD2, PKHD1, TMEM67, TSC1, TSC2, TTC21B, UMOD, VHL, WDR19, ALG8, ALG9, CEP290, COL4A4, ETFA, FLCN, LRP5, NOTCH2, PAX2, PMM2, and SEC61A1. Paired-end (PE) sequencing was conducted with a high-output flow cell involving 300 cycles of PE (150 bp × 2) on a NextSeq500 instrument (Illumina, San Diego, CA, USA) at a CAP-certified medical laboratory (Green Cross Genome, Yongin, Republic of Korea). The trimmed raw sequence was aligned to the human reference genome (hg19; NCBI build GRCh37). Data were analyzed following the Genome Analysis Tool Kit best practice pipeline workflow (https://gatk.broadinstitute.org/hc/en-us (accessed on 12 April 2023), which encompasses processes such as base calling, base alignment, variant calling, annotation, and quality control reporting. Medical laboratory geneticists independently reviewed the interpretation of sequence variants, adhering to the standards and guidelines established by the Joint Consensus Recommendation of the American College of Medical Genetics and Genomics and Association for Molecular Pathology to ensure accuracy [17]. The mean depth of coverage was 1014×, and 100% of the bases had coverage greater than the 10× variant interpretation criteria.

The sequencing of the *PKD1* gene revealed a heterozygous pathogenic variant, c.165_171del (p.Leu56ArgfsTer15), which genetically confirmed the diagnosis of ADPKD type 1. This mutation is rare and has not been previously reported in the general population (gnomAD and KRGDB—The Genome Aggregation Database and Korean Reference Genome Database). Additionally, it is predicted to create a frameshift and premature stop codon via a 7 bp deletion (a truncating mutation). This mutation has been reported in patients with ADPKD and is classified as a pathological variant in the ClinVar database.

The patient was started on telmisartan (40 mg) for hypertension, along with lifestyle modifications. The target blood pressure was 130/80 mmHg, and an outpatient visit once every 6 months was recommended.

Genetic counselors provided the patient with essential information about reproductive options, including preimplantation genetic diagnoses and assisted reproductive technologies, which can significantly reduce the risk of transmitting genetic conditions to future children.

## 3. Discussion

Cystic kidney disease encompasses a group of disorders characterized by the formation of fluid-filled sacs, or cysts, in or around the kidneys. The two major types of cystic kidney disease are acquired cystic kidney disease and polycystic kidney disease. Polycystic kidney disease is a genetic disorder that leads to the development of numerous cysts in the kidneys. Polycystic kidney disease may develop due to several genetic disorders, such as ADPKD, autosomal recessive polycystic kidney disease, autosomal dominant tubulointerstitial kidney disease, and nephronophthisis [1]. As polycystic kidney disease is a genetic disease, genetic testing is critical for the accurate diagnosis of polycystic HSK. Acquired cystic kidney disease is one of the conditions that needs to be ruled out in order to diagnose cystic kidney disease. Since this patient did not undergo hemodialysis, acquired cystic kidney disease could be excluded.

The horseshoe kidney is frequently linked to specific chromosomal disorders. The most prevalent disorders associated with horseshoe kidney are Patau syndrome (Trisomy 13), Turner syndrome, Edwards syndrome (Trisomy 18), and Down syndrome (Trisomy 21) [18]. The clinical signs of Turner syndrome, Edwards syndrome, Patau syndrome, and Down syndrome were not found in this patient.

To date, more than 20 instances of polycystic horseshoe kidney have been documented [16,19]. However, genetic tests were not used to diagnose the majority of them. In this study, we reported on a patient with ADPKD and horseshoe kidney after conducting genetic testing. We found that there was *PKD1* gene mutation.

In patients with ADPKD, water fills the cysts, enlarging the kidney, leading to pressure on the renal parenchyma and decreased renal function [20]. *PKD1* (16p13.3) and *PKD2* (4q21) are the two most well-known causative gene mutations in ADPKD [21]. Additionally, GANAB and HNF1β mutations may lead to mild polycystic kidney disease [22]. There was a PKD1 gene anomaly in this patient.

Cysts may form not only in the kidneys but also in the liver, pancreas, seminal vesicles, arachnoid membrane, and nerve root sheath (spinal meningeal diverticula) [23]. As ADPKD can be associated with hernia, cerebral aneurysm, or mitral valve prolapse, systemic complications can also occur. It is a disease inherited in an autosomal dominant fashion, and generations of families may suffer from ADPKD. However, ADPKD due to a de novo mutation occurs in approximately 10% of cases [24]. The patient reported on here did not have a family history of cerebral aneurysm or subarachnoid hemorrhage. The magnetic resonance angiography of the brain revealed no aneurysms.

HSK is commonly associated with skeletal, cardiovascular, and nervous system abnormalities [25]. It is often found in patients with Edwards syndrome, Turner syndrome, or neural tube defects [26]. Additionally, urinary tract malformations such as duplicate ureters, ectopic ureterocele, vesicoureteral reflux, polycystic kidney disease, hypospadias, cryptorchidism, and seminal megavesicles [27] may be accompanied by genital abnormalities in patients with HSK [28]. Kidney stones, urinary tract infections, and hydronephrosis may occur; however, the renal function is usually normal [29]. In the present case, there were no urinary tract malformations except polycystic kidney disease.

Given the patient’s young age and family history, it was crucial to discuss the potential risks of passing on genetic conditions associated with early-onset end-stage kidney disease. We included information on the inheritance patterns of relevant genetic mutations, such as those associated with ADPKD, which have a 50% chance of being transmitted to offspring [30]. Additonally, we explained genetic counseling is important in family planning. Genetic counselors can provide essential information about reproductive options, including preimplantation genetic diagnosis and assisted reproductive technologies [31,32]. We emphasized that genetic counseling involves a comprehensive discussion of the implications of genetic findings for family planning decisions.

In this case, the patient’s genetic testing results provided critical insights that directly influenced both clinical decision-making and counseling strategies The genetic counseling elucidated the implications of the identified mutations and potential risks of polycystic kidney disease. This genetic counseling was particularly crucial, given the patient’s young age, as it enabled him and his family to make decisions regarding family planning.

HSK disease is a congenital urinary tract malformation and multifactorial disorder that occurs due to the interaction of genetic susceptibility, epigenetic factors, and environmental factors [33]. Additional research is needed to determine the genetic susceptibility of patients with HSK and to identify a genetic link between polycystic kidney disease and HSK disease. Further investigation regarding the genetic links of a patient is also necessary.

Polycystic horseshoe kidney disease with known genetic defects is rare. Alobaili et al. [15] reported a case of polycystic horseshoe kidney associated with the heterozygous variant c.11315_11316insA (Ala3773Profs*59). However, there is the possibility of missing literature or unpublished cases. In this patient, the genetic defect was c.165_171del (p.Leu56ArgfsTer15). Although mutations in *PKD1* have been reported previously, this case report enriches their database.

## 4. Conclusions

In this case report, we suggest that genetic testing becomes the key approach to the diagnosis of ADPKD with horseshoe kidney. Additionally, this approach offers the benefit of avoiding the possibility of the condition being mistakenly diagnosed or diagnosed late due to its uncommon occurrence and nonspecific symptoms.

## Figures and Tables

**Table 1:** Clinical parameters of patient.

Parameter	Details
Age	24 years
Gender	Male
Family history	Positive for ADPKD (father and uncle affected) The other five male brothers of the patient’s father were not affected
Symptoms	No flank pain No gross hematuria, proteinuria, or albuminuria Hypertension
Blood pressure	150/90 mmHg
Physical examination	No palpable abdominal kidney
Laboratory examination	Serum creatinine 0.85 mg/dL
	Estimated GFR 122 mL/min/1.7 m^2^ Creatinine clearance 104 mL/min Urea clearance 101 mL/min Blood urea nitrogen 16 mg/dL Aspartate aminotransferase 28 U/L Alanine aminotransferase 38 U/L Total bilirubin 0.75 mg/dL Albumin 4.9 g/dL Hb 15.0 g/dL U/A RBC 0-2/HPF, Protein (-), Urine protein-to-creatinine ratio 79 mg/g Urine albumin-to-creatinine ratio 14 mg/g
Kidney volume	Right 542 mL, Left 440 mL
Genetic testing	PKD1 mutation (c.165_171del (p.Leu56ArgfsTer15))
Management plan Pedigree	Lifestyle modifications Antihypertensive medications with telmisartan 40 mg Regular monitoring of blood pressure and kidney function every 6 months 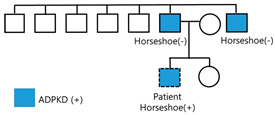

## Data Availability

The data presented in this study are available on request from the corresponding author.

## Abbreviations

The following abbreviations are used in this manuscript:

PKD1	polycystin 1
ADPKD	autosomal dominant polycystic kidney disease
PKD	polycystic kidney disease
HSK	horseshoe kidney
